# Topical Administration of Heat-Killed *Enterococcus faecalis* Strain KH2 Promotes Re-Epithelialization and Granulation Tissue Formation during Skin Wound-Healing

**DOI:** 10.3390/biomedicines9111520

**Published:** 2021-10-22

**Authors:** Hiromasa Tanno, Emi Kanno, Shiho Kurosaka, Yukari Oikawa, Takumi Watanabe, Ko Sato, Jun Kasamatsu, Tomomitsu Miyasaka, Shinyo Ishi, Miki Shoji, Naoyuki Takagi, Yoshimichi Imai, Keiko Ishii, Masahiro Tachi, Kazuyoshi Kawakami

**Affiliations:** 1 Department of Science of Nursing Practice, Tohoku University Graduate School of Medicine, 2-1 Seiryo-machi, Aoba-ku, Sendai 980-8575, Japan; ekanno@med.tohoku.ac.jp; 2 Department of Plastic and Reconstructive Surgery, Tohoku University Graduate School of Medicine, 2-1 Seiryo-machi, Aoba-ku, Sendai 980-8575, Japan; shihoko_5x2@yahoo.co.jp (S.K.); ishishinyoushi@yahoo.co.jp (S.I.); miki_shouji_0121@yahoo.co.jp (M.S.); takagi-prs@med.tohoku.ac.jp (N.T.); yo-imai@med.tohoku.ac.jp (Y.I.); tachi@med.tohoku.ac.jp (M.T.); 3 Department of Medical Microbiology, Mycology and Immunology, Tohoku University Graduate School of Medicine, 2-1 Seiryo-machi, Aoba-ku, Sendai 980-8575, Japan; yukari.oikawa.s1@dc.tohoku.ac.jp (Y.O.); ko-sato@med.tohoku.ac.jp (K.S.); ishii-k@med.tohoku.ac.jp (K.I.); kawakami@med.tohoku.ac.jp (K.K.); 4 Bio-Lab Co., Ltd., 2-1-3 Komagawa, Hidaka-shi 350-1249, Japan; t.watanabe@bio-ken.jp; 5 Department of Intelligent Network for Infection Control, Tohoku University Graduate School of Medicine, 2-1 Seiryo-machi, Aoba-ku, Sendai 980-8575, Japan; kasamatsu@med.tohoku.ac.jp; 6 Division of Pathophysiology, Department of Pharmaceutical Sciences, Faculty of Pharmaceutical Sciences, Tohoku Medical and Pharmaceutical University, Sendai 981-8558, Japan; t-miya13@tohoku-mpu.ac.jp

**Keywords:** wound-healing, *Enterococcus faecalis* KH2, angiogenesis

## Abstract

Lactic acid bacteria (LAB) are known to have beneficial effects on immune responses when they are orally administered as bacterial products. Although the beneficial effects of LAB have been reported for the genera *Lactobacillus* and *Lactococcus*, little has been uncovered on the effects of the genus *Enterococcus* on skin wound-healing. In this study, we aimed to clarify the effect of heat-killed *Enterococcus faecalis* KH2 (heat-killed KH2) strain on the wound-healing process and to evaluate the therapeutic potential in chronic skin wounds. We analyzed percent wound closure, re-epithelialization, and granulation area, and cytokine and growth factor production. We found that heat-killed KH2 contributed to the acceleration of re-epithelialization and the formation of granulation tissue by inducing tumor necrosis factor-α, interleukin-6, basic fibroblast growth factor, transforming growth factor (TGF)-β1, and vascular endothelial growth factor production. In addition, heat-killed KH2 also improved wound closure, which was accompanied by the increased production of TGF-β1 in diabetic mice. Topical administration of heat-killed KH2 might have therapeutic potential for the treatment of chronic skin wounds in diabetes mellitus. In the present study, we concluded that heat-killed KH2 promoted skin wound-healing through the formation of granulation tissues and the production of inflammatory cytokines and growth factors.

## 1. Introduction

Wounds are categorized as acute or chronic based on the time it takes them to heal. Acute wounds generally heal within 2 weeks whereas chronic wounds can take several months or longer. Acute wound-healing in the skin moves through inflammation, proliferation, and remodeling phases [1]. In the inflammatory phase, keratinocytes, fibroblasts, and leukocytes produce inflammatory cytokines, such as interleukin (IL)-1β, IL-6, and tumor necrosis factor (TNF)-α, which are important for leukocyte recruitment and protection against infection [2,3]. In the proliferative phase, numerous growth factors, such as transforming growth factor (TGF)-β, epidermal growth factor (EGF), vascular endothelial growth factor (VEGF), and basic fibroblast growth factor (bFGF), are essential for the formation of granulation tissue, angiogenesis, and epithelialization, which play critical roles in the healing process [3,4]. In contrast, skin wound-healing in immunocompromised hosts, such as those with diabetes mellitus, are frequently delayed due to a prolonged inflammatory phase, leading to chronic and intractable diseases [5,6].

Patients with chronic wounds, including diabetic ulcers and pressure ulcers, suffer from many problems, such as an increased risk of infection, poor blood supply to wound tissues, insufficient angiogenesis, and impaired leukocyte function. Although multiple wound therapies have been developed, such as debridement, negative pressure wound therapy with instillation and dwelling (NPWTi-d), and treatment with antibacterial dressing, many problems related to chronic wounds remain unsolved and continue to incur massive medical expenses. Improvement in the blood supply and leukocyte function is required to assist patients with chronic wounds.

Lactic acid bacteria (LAB), which are Gram-positive bacteria, are well known to have beneficial effects on the regulation of immune responses and host protection against microbial infection [7,8,9]. Generally, LAB are classified as follows: *Lactobacillus* spp., *Lactococcus* spp., *Enterococcus* spp., and others [10]. The effects of LAB on skin wound-healing have been reported using *Lactococcus* spp. and *Lactobacillus* spp. For example, oral treatment with *L. reuteri* leads to the acceleration of wound closure and the increased deposition of collagen [11]. As part of the antimicrobial defense, the administration of *L. plantarum* at the burn-injured site contributes to improvement in sepsis and survival rates caused by infection with *Pseudomonas aeruginosa* [12]. Although it remains to be elucidated how the administration of *Enterococcus* spp. affects the process of wound-healing in skin, treatment with heat-killed *E. faecalis* strains EC-12 and KH2 was reported to induce the production of inflammatory cytokines by macrophages and to activate natural killer (NK) cells and cytotoxic T cells, respectively [13,14]. In addition, several studies revealed that LAB promote leukocyte function and increase the production of VEGF, one of the most important mediators of angiogenesis [13,14,15]. In the present study, we hypothesized that the topical application of heat-killed *E. faecalis* KH2 at the wound site could contribute to resolving these chronic wound problems.

Against this background, the current study was performed to clarify the effects of the topical administration of heat-killed *E. faecalis* KH2 (heat-killed KH2) on the wound-healing process using mouse models of acute skin wound-healing and chronic wound-healing under a diabetes mellitus condition.

## 2. Materials and Methods

### 2.1. Animals

A total of 125 (*n* = 59 mice for vehicle group, *n* = 66 mice for KH2 group) male C57BL/6J mice were purchased from CLEA Japan (Tokyo, Japan) and were 7 to 8 weeks old. All mice were bred in a specific pathogen–free environment in the Institute for Animal Experimentation, Tohoku University Graduate School of Medicine (Sendai, Japan). The conditions of the breeding room were as follows: room temperature, 20–26 °C; humidity, 40–60%; light/dark cycle, 12 h; and *ad libitum* availability of food and water. Purchased mice were acclimatized for at least a week before wounding. We took the utmost care to alleviate any pain and suffering in mice during the experiments.

### 2.2. Wound Creation and Tissue Collection

Mice were anesthetized with an intraperitoneal injection of 40 mg/kg sodium pentobarbital (Somnopentyl, Kyoritsu Seiyaku Corporation, Tokyo, Japan), and sustained by inhalation anesthesia of isoflurane (Isoflurane, Mairan Pharma, Osaka, Japan). The dorsal hair was shaved to fully expose the skin, which was then rinsed with 70% ethanol. Four full-thickness wounds extending to the panniculus carnosus were created using a 6-mm-diameter biopsy punch (Biopsy Punch, Kai Industries Co., Ltd., Gifu, Japan) under sterile conditions. The injured areas were covered with a polyurethane film (Tegaderm Transparent Dressing, 3M Health Care, Saint Paul, MN, USA) and an elastic adhesive bandage (Hilate, Iwatsuki, Tokyo, Japan) for an occlusive dressing. At various time points, mice were sacrificed and the wound tissue was collected by excising a 1-cm-square section of skin using scissors and a surgical knife. The tissue was then processed for histopathological analysis and measurement of cytokine concentrations.

### 2.3. Preparation of Heat-Killed E. faecalis KH2

*E. faecalis* KH2 (International Patent Organism Depositary, Japan, NITE P-14444; GenBank Accession number, AB534553), isolated from a human fecal sample, was stored at Bio-Lab Co., Ltd. (Saitama, Japan) [16]. *E. faecalis* KH2 was grown aerobically overnight at 37 °C in MRS broth (Difco, Detroit, MI, USA), washed centrifugally with distilled water at 10,000× *g* for 3 min, and then the bacteria were collected by centrifugation. The bacterial suspension in distilled water was heated at 105 °C for 30 min using an autoclave (HV-25ⅡLB; Hirayama Manufacturing Corp., Saitama, Japan). To increase the water dispersibility of the heat-killed KH2, this heat-killed bacteria was treated using a high-pressure homogenizer (ECONIZER LABO-01; Sanmaru Machinery Co., Ltd., Shizuoka, Japan) at 15 MPa, and an equal amount of dextrin (NSD300; San-ei Sucrochemical Co., Ltd., Aichi, Japan) was added. Heat-killed KH2 and dextrin were diluted with normal saline to a concentration of 100 mg/mL.

### 2.4. Heat-Killed KH2 Treatment of Wounds

Wounds were created in accordance with the method described above. Immediately after wounding, a 5 μL suspension of heat-killed KH2 (500 μg) or equal volume of dextrin as a vehicle control was applied to the base of the wounds in mice.

### 2.5. Measurement of the Wound Area

Morphometric analysis was performed on digital images using a digital camera (CX4, Ricoh, Tokyo, Japan). After the wounds were created, photographs were taken of each wound before dressing. At various time points, the polyurethane films were gently removed from the sacrificed mice, and the wounds were photographed. The wound area was quantified by tracing its margin and calculating the pixel area using AxioVision imaging software, Release 4.6 (Carl Zeiss Micro Imaging Japan, Tokyo, Japan). Wound-healing was evaluated as the percent wound closure, which was calculated using the following formula: % wound closure = (1 − wound area at the indicated time point/wound area on day 0) × 100.

### 2.6. Histopathology and Immunohistochemistry

The wounded tissues were fixed with 4% paraformaldehyde-phosphate buffer solution and embedded in paraffin as previously described [17,18,19]. Sections were harvested from the central portion of the wound and stained with hematoxylin-eosin (HE) according to the standard method. The extent of re-epithelialization of each wound was measured in these HE-stained sections by measuring the distance from the normal wound margin to the edge of the epithelium. The re-epithelialization index was determined based on the percentage of new epithelium present in the total wound. Granulation area was determined on HE-stained sections. For immunohistochemistry, the sections were stained with anti-CD31 Ab (1:600 dilution; Santa Cruz Biotechnology Inc., Dallas, TX, USA) after endogenous peroxidase and nonspecific binding were blocked. They were then incubated with peroxidase-conjugated secondary antibodies (Histofine^®^ Simple Stain MAX-PO, Nichirei Bioscience, Tokyo, Japan). Control sections were treated with non-immune IgG in place of any of the first antibodies. Five random fields (0.2 mm^2^ in area each) in granulation tissue were selected and analyzed under 400× magnification (Olympus BH-2, Olympus Optical Co., Ltd., Tokyo, Japan).

### 2.7. Measurement of Cytokine and Growth Factor Concentrations

The wound tissues were homogenized with normal saline, and concentrations of cytokines and growth factors in the supernatants were measured by enzyme-linked immunosorbent assay (ELISA) kits (BioLegend, San Diego, CA, USA, for TNF-α, IL-1β, and IL-6; R&D Systems, Minneapolis, MN, USA, for bFGF, TGF-β, VEGF, and EGF). The results were expressed as values per wound.

### 2.8. Induction of Diabetes Mellitus

Diabetic mice were established by intraperitoneal injection of 100 mg/kg streptozotocin (STZ) (Santa Cruz Biotechnology, Inc.) dissolved in 5 mM sodium citrate buffer (pH 4.5) after one night starvation. After 14 days, the blood glucose levels were higher than 250 mg/dL, and the mice were considered to have diabetes.

### 2.9. Statistical Analysis

Data were analyzed using JMP^®^ Pro 15 0.0 software (SAS Institute Japan, Tokyo, Japan). Data are expressed as the mean ± standard deviation (SD). Differences between groups were examined for statistical significance using Welch’s *t*-test. A *p* value less than 0.05 was considered significant.

## 3. Results

### 3.1. Accelerated Skin Wound-Healing by Treatment with Heat-Killed KH2

**Figure 1:**
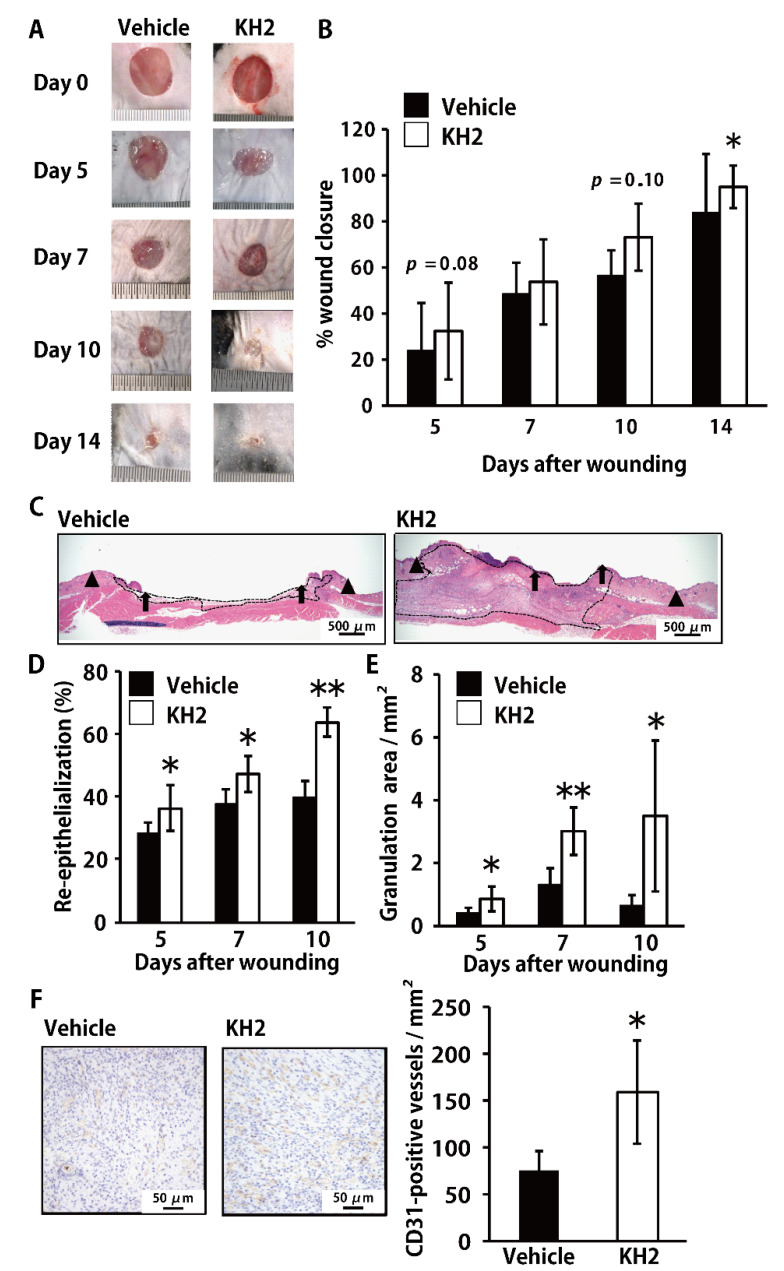
Effect of heat-killed KH2 administration on skin wound-healing. Wounds were created on the backs of vehicle or heat-killed KH2-treated mice. Photographs (**A**) were taken, and the percentages of wound closure (**B**) were evaluated on days 5, 10, and 14 (*n* = 8–20 wounds). (**C**) Representative histological views of skin wounds on day 10 are shown. Arrowheads, arrows, and the dotted line indicate the original wound edges, re-epithelialized leading edges, and granulation area, respectively. The wound edges were determined as the border between the normal epithelium and the thick proliferative epithelium. (**D**) Time-course changes in the re-epithelialization ratio after wound creation are shown (*n* = 4–6 wounds). (**E**) Time-course changes in the granulation tissue area after wound creation are shown (*n* = 4–6 wounds). (**F**) Micrographs show CD31-positive cells stained with anti-CD31 antibody on day 10. The vascular density/mm^2^ was determined by counting the number of positive vessels within 5 visual fields (*n* = 4–6 wounds). Each column represents the mean ± standard deviation. The results are representative of at least two independent experiments. * *p* < 0.05, ** *p* < 0.01.

To address the possible promotion of wound-healing by heat-killed KH2, we examined the effect of treatment with this bacterium on the wound-healing process in skin. In our preliminary experiments, heat-killed KH2 showed the highest promotion of wound-healing at 500 μg/wound compared to the effect at 5 or 50 μg/wound (data not shown). Therefore, we chose 500 μg/wound to analyze the effect of heat-killed KH2 on skin wound-healing in the current study. As shown in Figure 1A,B, wild-type (WT) mice treated with heat-killed KH2 showed significant acceleration of wound closure on day 14 after wounding compared with mice treated with vehicle control. Although treatment with heat-killed KH2 showed a trend toward accelerated wound closure, no significance was observed in vehicle-treated controls on days 5 and 10 (Figure 1B). In heat-killed KH2-treated mice, the re-epithelialization rate was significantly increased compared with vehicle-treated mice on days 5, 7, and 10 (Figure 1C,D). Additionally, the granulation area was significantly increased in heat-killed KH2-treated mice compared with vehicle-treated mice on days 5, 7, and 10 (Figure 1C,E). As alternate indicators of wound-healing, we also evaluated CD31 expression in the wounded tissues, which indicate vascularization. As shown in Figure 1F, CD31-positive vessel counts were markedly increased in heat-killed KH2-treated mice compared with vehicle-treated mice on day 10.

### 3.2. Enhanced Synthesis of Inflammatory Cytokines by Heat-Killed KH2 Treatment

**Figure 2:**
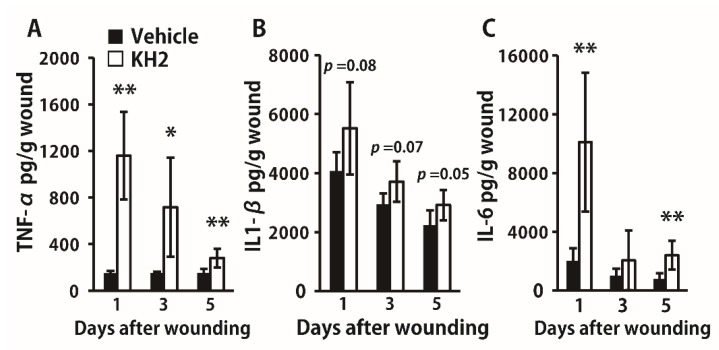
Effect of heat-killed KH2 administration on the production of TNF-α, IL-1β, and IL-6. (**A**) Production of TNF-α in the homogenates of wound tissues was measured on days 1, 3, and 5 after wound creation (*n* = 5–6 mice). (**B**) Production of IL-1β in the homogenates of wound tissues was measured on days 1, 3, and 5 after wound creation (*n* = 5–6 mice). (**C**) Production of IL-6 in the homogenates of wound tissues was measured on days 1, 3, and 5 after wound creation (*n* = 5–6 mice). Each column represents the mean ± standard deviation. The results are representative of at least two independent experiments. * *p* < 0.05, ** *p* < 0.01.

Next, we evaluated how heat-killed KH2 treatment affected the synthesis of inflammatory cytokines in the wounded tissues. TNF-α was significantly more highly synthesized in the heat-killed KH2-treated group than in the vehicle-treated group on days 1, 3, and 5 (Figure 2A). IL-1β synthesis tended to be higher in the heat-killed KH2-treated group than in the vehicle-treated group (Figure 2B). Additionally, IL-6 synthesis was also increased in mice treated with heat-killed KH2 compared with vehicle-treated mice on days 1 and 5 (Figure 2C).

### 3.3. Enhanced Synthesis of Growth Factors by Heat-Killed KH2 Treatment

**Figure 3:**
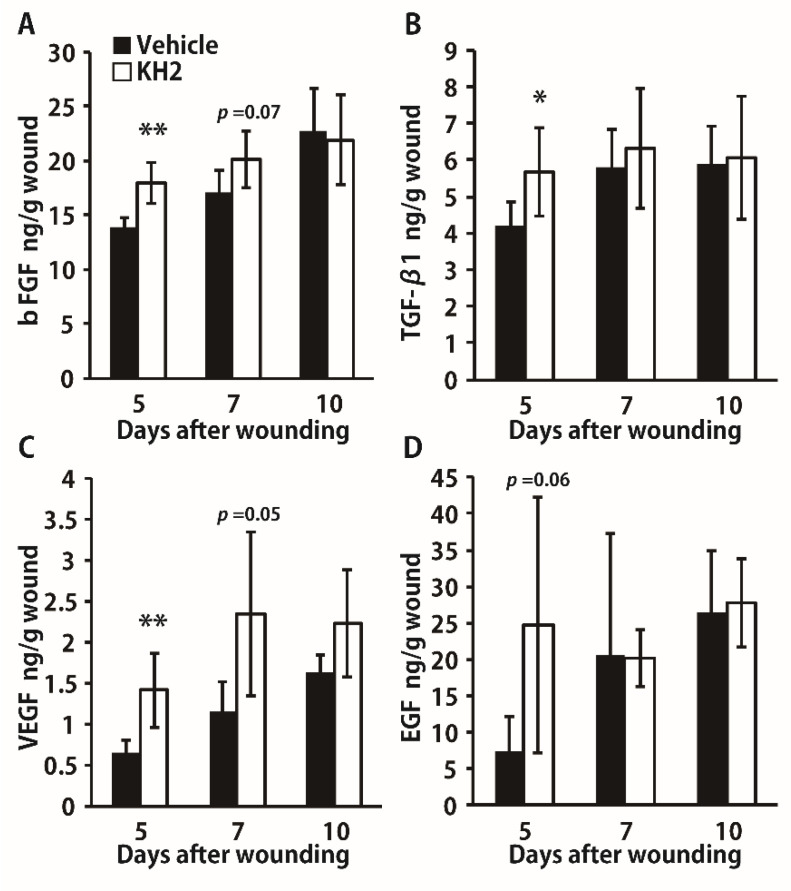
Effect of heat-killed KH2 administration on the production of bFGF, TGF-β1, VEGF, and EGF. (**A**) Production of bFGF in the homogenates of wound tissues was measured on days 5, 7, and 10 after wound creation (*n* = 5–6 mice). (**B**) Production of TGF-β1 in the homogenates of wound tissues was measured on days 5, 7, and 10 after wound creation (*n* = 5–6 mice). (**C**) Production of VEGF in the homogenates of wound tissues was measured on days 5, 7, and 10 after wound creation (*n* = 5–6 mice). (**D**) Production of EGF in the homogenates of wound tissues was measured on days 5, 7, and 10 after wound creation (*n* = 5–6 mice). Each column represents the mean ± standard deviation. The results are representative of at least two independent experiments. * *p* < 0.05, ** *p* < 0.01.

To further confirm the effects of heat-killed KH2, we examined the synthesis of growth factors after this treatment. As shown in Figure 3A–C, production of bFGF, VEGF, and TGF-β1 was significantly increased in mice treated with heat-killed KH2 compared to vehicle-treated mice on day 5 after wounding. In addition, the synthesis of bFGF and VEGF was higher in the heat-killed KH2-treated group on day 7, although the difference was not statistically significant. As shown in Figure 3D, EGF synthesis was not markedly different between mice treated with heat-killed KH2 and vehicle-treated mice.

### 3.4. Improved Wound Closure and TGF-β1 Synthesis in STZ-Induced Diabetic Mice by Heat-Killed KH2 Treatment

**Figure 4:**
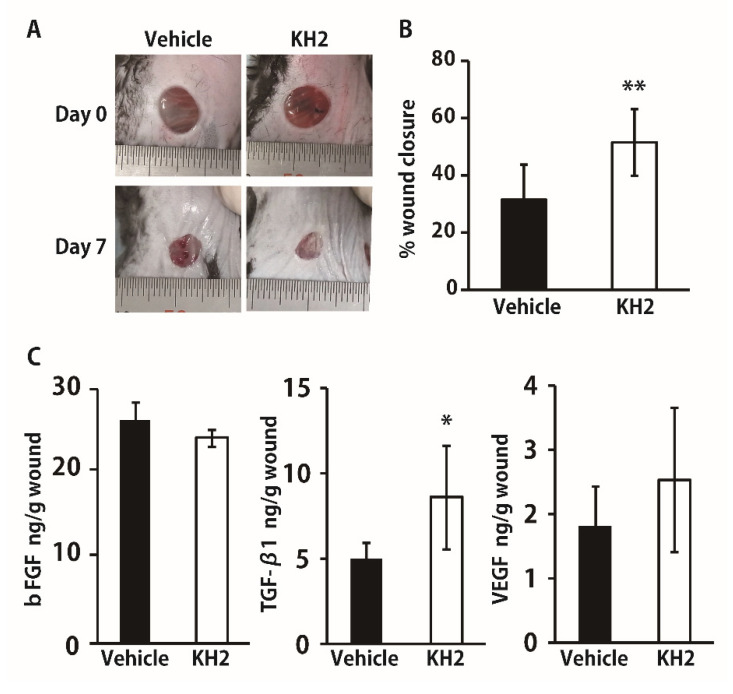
Effect of heat-killed KH2 administration on skin wound-healing in diabetic mice. Wounds were created on the backs of STZ-induced diabetic mice that were treated with vehicle or heat-killed KH2. (**A**) Representative photographs of wounds on days 0 and 7. (**B**) Percentage of wound closure was evaluated on day 7 (*n* = 12–14 wounds), (**C**) Production of bFGF, TGF-β1, and VEGF in the homogenates of wound tissues was measured on day 7 after wound creation (*n* = 6–7 mice). Each column represents the mean ± standard deviation. The results are representative of at least two independent experiments. * *p* < 0.05, ** *p* < 0.01.

We hypothesized that after treatment with heat-killed KH2, growth factors may be involved in the improvement of wounded tissues under the diabetic condition that may lead to promoted wound-healing. To address this possibility, we examined the effect of heat-killed KH2 treatment on wound closure and growth factor synthesis in the wounded skin tissues in diabetic mice caused by STZ injection. Because some studies reported that diabetic mice show slower growth factor production than normal mice, which leads to delayed wound-healing [20,21], we conducted the analysis 7 days after wounding, a later time point than in normal mice. As shown in Figure 4A,B, treatment with heat-killed KH2, but not with vehicle, significantly promoted wound-healing. In addition, the same treatment significantly enhanced the production of TGF-β1, but not of bFGF and VEGF, under the diabetic condition (Figure 4C).

## 4. Discussion

In the present study, we demonstrated for the first time that treatment with heat-killed *E faecalis* KH2 caused a significant acceleration of skin wound-healing, which was associated with increased re-epithelialization and granulation tissue formation. In addition, the same treatment also promoted wound closure with enhanced production of TGF-β1 in a chronic wound model with diabetes mellitus.

In the current study, the results of re-epithelialization and wound closure differed. Several studies investigating skin wound-healing showed different results for wound closure and re-epithelialization, similar to ours [22,23,24]. It may be more difficult to distinguish newly extended epithelium in macroscopic observations such as wound closure than in microscopic observations using HE-stained sections. Further investigations are necessary to address this issue.

In earlier studies [2,3], proinflammatory cytokines such as TNF-α and IL-6 were reported to play critical roles in the accumulation of inflammatory leukocytes in skin wounded tissue. Previously, we demonstrated that TNF-α treatment resulted in the acceleration of skin wound-healing [25]. Furthermore, previous investigators revealed that IL-6-deficient mice exhibited delayed wound-healing responses, suggesting the critical role of IL-6 in the wound-healing process in skin [26,27]. In agreement with these previous findings, in the current study, the increased production of TNF-α and IL-6 was associated with accelerated wound-healing caused by treatment with heat-killed KH2. Collectively with these findings, these proinflammatory cytokines are suggested to be highly involved in the progression of wound-healing processes in skin.

In the current study, treatment with heat-killed KH2 significantly increased the production of TNF-α and IL-6, but not of IL-1β. The production of proinflammatory cytokines, including TNF-α, IL-1β, and IL-6, is regulated by the NF-κB signaling pathway [28]. In addition, secretion of the active form of IL-1β requires activation of the NLRP3-inflammasome signaling pathway, which is unlike that for other cytokines [29]. This additional requirement of the signaling pathway could account for the distinct effects of heat-killed KH2 on the production of proinflammatory cytokines.

Various growth factors, including VEGF, TGF-β, and bFGF, have been reported to play important roles in skin wound-healing. These growth factors contribute to the progression of re-epithelialization, angiogenesis, and granulation tissue formation [3,4]. In fact, it is well known that platelet-rich plasma (PRP), an effective endogenous therapeutic strategy for chronic wounds, contains these growth factors, which accelerate this process [30,31,32]. In the present study, heat-killed KH2 treatment resulted in accelerated re-epithelialization, granulation tissue formation, and angiogenesis, which was accompanied by the increased production of these growth factors in wounded skin tissues. In previous studies, the production of VEGF, TGF-β, and bFGF was reported to be promoted by proinflammatory cytokines, including TNF-α and IL-6 [33,34,35]. These results suggest that the enhanced production of VEGF, TGF-β1, and bFGF caused by heat-killed KH2 treatment may be due to the up-regulation of TNF-α and IL-6 production, which may lead to the promotion of re-epithelialization, granulation tissue formation, and angiogenesis.

Earlier studies reported that STZ-induced diabetic mice exhibited impaired wound closure and angiogenesis that was associated with the reduced production of growth factors, including TGF-β1 [20,36]. In the present study, we evaluated the effect of heat-killed KH2 treatment on the skin wound-healing process using STZ-induced diabetic mice and observed that wound closure was significantly accelerated and was accompanied by increased TGF-β1 production on day 7 after wound creation. Our results suggest that heat-killed KH2 administration may be effective in improving delayed skin wound-healing under the diabetic condition. In the present study, however, we only analyzed the wound closure rate and growth factor production on day 7 after wound creation. Further investigations that include similar research points at different time points with distinct indicators in the wound-healing processes are necessary to define the beneficial effects of heat-killed KH2 treatment on delayed skin wound-healing under the diabetic condition.

The precise mechanism by which the immune system recognizes LAB at the wounded sites remains to be elucidated. Heat-treated LAB release cytoplasmic components (including DNA) and cell wall components (including peptidoglycans, lipoteichoic acids). These heat-killed LAB components are also recognized by immune systems and are able to stimulate immune cells [37]. Inoue and co-workers demonstrated that immune cells are stimulated by heat-killed *E. faecalis* through recognition via Toll-like receptor (TLR) 9 in vitro [13]. This pattern recognition receptor is known to sense unmethylated oligo DNA containing the canonical CpG motif, which leads to the production of TNF-α and IL-6 by activated immune cells [38,39]. During the wound-healing process, skin resident cells (including keratinocytes and fibroblasts) [40,41] and non-resident cells (including macrophages, neutrophils, and T cells) express TLR9 [42,43,44]. In addition, the signaling triggered via TLR9 contributes to the accelerated skin wound-healing. Specifically, mice triggered by CpG ODN, a TLR9 ligand, exhibited accelerated skin wound-healing together with increased production of TNF-α, IL-6, VEGF, and TGF-β in the wounded tissues [45,46], similar to the heat-killed KH2-treated mice in the current study. Collectively, these findings suggest that TLR9-expressing cells may recognize heat-killed KH2 for the induction of inflammatory cytokine and growth factor production and the promotion of skin wound-healing responses.

Several studies reported that the beneficial effects of heat-killed LAB observed in hosts are equivalent to those of live LAB [47,48,49]. In comparison with live LAB, heat-killed LAB has some advantages, such as a lowered risk of transfer of antibiotic resistance to other bacteria in the surrounding environment [37]. Thus, the topical administration of heat-killed KH2 may be clinically applicable to the treatment of chronic skin wounds, especially in patients with underlying conditions such as diabetes mellitus, a major cause of impaired wound-healing. Although the possibility of excessive inflammation, such as systemic inflammatory response syndrome, could not be denied, this treatment may be advantageous, as shown by the lowered risk of sepsis caused by this bacterium.

Thus, the lactic acid bacterium used in the present study may be a novel therapeutic option for the treatment of chronic intractable skin wounds in diabetic patients.

## 5. Conclusions

In conclusion, the present study demonstrated that treatment with the heat-killed KH2 strain of *E. faecalis* promoted skin wound-healing through the production of inflammatory cytokines, which may be involved in enhanced growth factor production. In addition, the increased production of growth factors may lead to the acceleration of re-epithelialization, granulation tissue formation, and angiogenesis. Importantly, the promoted skin wound-healing responses induced by this treatment were also observed in diabetic mice, in which these responses are well known to be impaired in clinical and experimental situations.

## Data Availability

Not applicable.
